# Combining Irradiation and Biological Control against Brown Marmorated Stink Bug: Are Sterile Eggs a Suitable Substrate for the Egg Parasitoid *Trissolcus japonicus*?

**DOI:** 10.3390/insects14070654

**Published:** 2023-07-22

**Authors:** Gerardo Roselli, Gianfranco Anfora, Raffaele Sasso, Livia Zapponi, Sergio Musmeci, Alessia Cemmi, David Maxwell Suckling, Kim Alan Hoelmer, Claudio Ioriatti, Massimo Cristofaro

**Affiliations:** 1 Center Agriculture, Food and Environment (C3A), University of Trento, 38098 San Michele all’Adige, Italy; gerardo.roselli@unitn.it; 2 Technology Transfer Centre, Fondazione Edmund Mach, 38098 San Michele all’Adige, Italy; 3 Biotechnology and Biological Control Agency (BBCA Onlus), 00123 Rome, Italy; 4 Laboratory SSPT-BIOAG-SOQUAS, ENEA C.R. Casaccia, 00123 Rome, Italy; 5 Institute of BioEconomy, National Research Council of Italy, 38098 San Michele all’Adige, Italy; 6 Laboratory FSN-FISS-SNI, ENEA C.R. Casaccia, 00123 Rome, Italy; 7 Formerly the New Zealand Institute for Plant and Food Research Ltd., PB 4704, Christchurch 8011, New Zealand; 8 Formerly School of Biological Sciences, University of Auckland, Auckland 1072, New Zealand; 9 USDA, Agriculture Research Service, Beneficial Insects Introduction Research Unit, Newark, DE 19713, USA

**Keywords:** SIT, biological control, *Trissolcus japonicus*, *Halyomorpha halys*, IPM, sentinel eggs

## Abstract

**Simple Summary:**

Halyomorpha halys is an alien stink bug species native to south-eastern Asia, and is now widely distributed and very invasive worldwide. Management of this pest by chemical insecticides is not very effective because of the high mobility of the pest species and its innate ability to develop resistance to most of the synthetic insecticides applied. For this reason, classical biological control has been considered, with attention focused on co-evolved egg parasitoids, which oviposit and complete their larval development within a single egg of the host. The detection and collection of the egg parasitoid species is often conducted by exposing newly oviposited egg-masses of the stink bug pest in the field for a few days as sentinels, which can then be parasitized by the egg parasitoids. In newly invaded areas, limiting factors in the use of sentinel eggs include the short amount of time that they are a suitable substrate for the oviposition of the egg parasitoid and the risk of the unintentional release of additional pests from unparasitized eggs. A valid potential alternative is to use sterile sentinel eggs of the pest species. This study focused on the evaluation of the performance of three types of sterile sentinel eggs for the oviposition and the larval development of the egg parasitoid Trissolcus japonicus.

**Abstract:**

The brown marmorated stink bug (BMSB), *Halyomorpha halys*, is a phytophagous invasive pest native to south-eastern Asia, and it is now distributed worldwide. This species is considered to be one of the most damaging insect pests in North America and in Europe. In agriculture, the predominant approach to managing BMSB is based on the use of insecticides, specifically pyrethroids and neonicotinoids. Unfortunately, the biology of the species and its facility to develop mechanisms of resistance to available pesticides has induced farmers and scientists to develop different, least-toxic, and more effective strategies of control. In a territorial area-wide approach, the use of a classical biological control program in combination with other least-toxic strategies has been given prominent consideration. Following exploratory surveys in the native range, attention has focused on *Trissolcus japonicus*, a small scelionid egg parasitoid wasp that is able to oviposit and complete its larval development in a single egg of *H. halys*. A common method for detecting egg parasitoids in the native range involves the placement of so-called ‘sentinel’ egg masses of the pest in the environment for a short period, which are then returned to the laboratory to determine if any of them are parasitized. Outside of the area of origin, the use of fertile sentinel eggs of the alien species may lead to the further release of the pest species; an alternative is to use sterile sentinel eggs to record the presence of new indigenous egg parasitoids or to detect the dispersal of alien species (in this case, *T. japonicus*) released in a new environment to control the target insect pest species. This study evaluated the performance of three types of sterile sentinel eggs as a suitable substrate for the oviposition and larval development of the egg parasitoid *T. japonicus* in a context of combining classical biological control with a Sterile Insect Technique (SIT) approach.

## 1. Introduction

The brown marmorated stink bug (BMSB) *Halyomorpha halys* (Stål) (Hemiptera: Pentatomidae) is a phytophagous insect native to Eastern Asia [1]. It has successfully invaded various regions, including Europe, North America [2,3], and the Caucasus [4]. Its potential for further expansion is substantial, particularly in the Southern Hemisphere [5,6]. Due to its wide host range, the BMSB species is considered an important pest species in the invaded areas, causing significant crop losses [3,7]. The predominant approach to managing BMSBs involves the use of insecticides, specifically pyrethroids and neonicotinoids along with other agronomic and biocontrol strategies [8,9,10]. However, broad-spectrum insecticides are a short-term solution, and their frequent use can induce insecticide resistance and can sometimes lead to secondary pest outbreaks [11,12]. On the other hand, the implementation of both indigenous and exotic natural biocontrol agents has shown promising medium- and long-term outcomes in controlling BMSBs [3,13,14,15]. In particular, the Asian egg parasitoid *Trissolcus japonicus* (Ashmead) (Hymenoptera: Scelionidae) has been identified as the most promising candidate for classical biological control of BMSBs [13,16] due to its relative host-specificity and high parasitization rate [17,18]. The presence of adventive populations of *T. japonicus* has been documented in the United States since 2014 [19], and it has been observed in Europe since 2017 [20,21]. In the United States, initiatives have been taken to facilitate the dispersal of the adventive populations of *T. japonicus* [22,23]. In Europe, meanwhile, Italy adopted a different approach, implementing a conventional classical biological control program by inoculating individuals in order to establish self-sustaining populations that would subsequently proliferate in response to the presence of the BMSB species [15].

In the context of an integrated pest management (IPM) strategy, combining classical biological control (CBC) and a sterile insect technique (SIT) may represent a synergistic and effective approach. SIT is considered a species-specific method, and it relies on the mass production, sterilization, and subsequent inundative release of predominantly sterile male insects [24]. The synergy between the SIT and the classical biological control approaches [25,26] further strengthens the opportunity to implement IPM or area-wide eradication programs for managing the BMSB species within its invasive range [27]. In recent years, indeed, there has been a notable surge in this—specifically, in the adoption of integrated biological control and SIT [28,29].

A pest management program that involves the use of biological control agents (BCAs) requires a good field monitoring system for their detection, which, for BMSBs, is based on sentinel eggs for egg parasitoids [30,31,32,33]. To minimize the risk of inadvertently introducing the pest into the environment by using fertile sentinel eggs, several alternatives were evaluated. Frozen BMSB egg masses at −80 °C were commonly used [14,34,35], although lower *T. japonicus* emergence was observed from the frozen BMSB eggs compared to fresh eggs [35]. Unfertilized and refrigerated eggs were then evaluated in substitution for frozen eggs, and they showed better performance in the emergence and development of *T. japonicus* [36,37].

Cristofaro et al. [38] proposed a new concept of using sterile sentinel eggs produced by SIT for the management of *Bagrada hilaris* (Burmister), which is another pentatomid pest species. Consequently, in the present study, besides the classical SIT approach, which is based on the release of sterile irradiated males, we compared the performance of two different types of sterile eggs—ones derived by the SIT and ones derived by the irradiation of fresh eggs—used as sentinel eggs for *T. japonicus.* The work was divided into three parts. The first two parts aimed to identify the correct dose of radiation needed to produce sterile eggs and sterile males of the BMSB species, while the third experiment evaluated the suitability of the eggs obtained by this process in comparison with eggs sterilized by refrigeration [37].

## 2. Materials and Methods

### 2.1. Insects and Rearing

The *Halyomorpha halys* colony originated from adults collected using traps in May 2018 from infested field sites in Rome, Italy (42°2′36.96″ N, 12°17′53.66″ E). Laboratory colonies of BMSBs were maintained in a temperature-controlled climatic chamber (27 ± 1 °C, photo-period L.D. = 16:8, RH = 70 ± 10%) in the entomology laboratories at the Italian National Agency for New Technologies, Energy and Sustainable Economic Development (ENEA), Rome (Italy). BMSBs were reared in cages (60 × 30 × 30 cm BugDorm^®^, Taichung, Taiwan) at a density of about 50–70 adults per cage, with approximately a 1:1 sex ratio. Insects had ad libitum access to water in a glass vial with a cotton plug, and they were fed with potted plants of *Phaseolus vulgaris* (L.) and *Vicia faba* (L.) as well as other fresh vegetables and fruits (green beans, carrots, apples, and kiwi). Senesced and dead host plants were removed and replaced with new plants weekly or as needed. Each week, the bottoms of all rearing cages were cleared of dead insects and debris (exuviae, loose soil, fallen leaves, and supplemental foods) and then scrubbed clean. Food was replenished three times per week. Females in the cages laid clusters of ~28 eggs on host plant leaves and on the top of the cages. The BMSB egg masses were collected daily (4 h after light activation in the climatic chamber), and 1-day-old or 2-day-old egg masses were used in the trials. The BMSB colony was regularly renewed or supplemented with part of the F1 generation of newly emerged adults and sometimes by field-collected adults. Plants within the cages were watered two to three times per week. The same treatment was performed to rear the juvenile forms.

A second BMSB colony was reared at the entomology laboratories of the Fondazione Edmund Mach (FEM), Trento (Italy), following the same protocol mentioned above. The colony originated from a mass trapping collection realized during autumn 2021 in fields near FEM (46°11′43″ N, 11°8′5″ E), which used live traps [39].

*Trissolcus japonicus* colonies were supplied by the Council for Agricultural Research and Agricultural Economy Analysis (CREA-DC, Florence, Italy), and they originated from the same population on which the risk assessment study was conducted [40]. They were reared at FEM facilities using BMSB egg masses obtained from colonies that were maintained specifically for this study. Following the same national protocol adopted for *T. japonicus* release [15], the individuals were placed in plastic tubes (VWR 50-mL centrifuge tubes, 525–0611) containing honey and maintained in climatic chambers at a temperature of 24 ± 1 °C, photoperiod L.D. = 16:8, and relative humidity (RH) of 60 ± 5%.

### 2.2. Egg Sterilization

Sterile eggs were obtained in three different ways:

(a) Irradiation of fresh eggs with gamma rays (irradiated eggs).
(b) Eggs obtained by mating BMSB fertile females with irradiated males (i.e., SIT eggs).
(c) Refrigeration of fresh BMSB eggs at 8 °C for a minimum of 14 days (refrigerated eggs, control).

#### 2.2.1. Irradiation

Egg masses of 24 and 48 h of post-oviposition age were exposed to γ radiation using cardboard tubes with a diameter of 9 cm. The radiation doses used were 0, 16, 24, 32, and 40 Gy, which were delivered using the Co-60 gamma facility at ENEA [41], with a dose rate of 175.03 Gy/h (2.92 Gy/min).

Male adult bugs were irradiated ~24 h after molting from fifth instar nymphs. Insects were exposed to radiation using the same protocol adopted for the egg masses, and the radiation doses used were 24 and 50 Gy.

#### 2.2.2. Egg Refrigeration

Refrigerated eggs that had been previously demonstrated as suitable as a substrate for the oviposition and development of the offspring of *T. japonicus* showed a higher level of parasitoid emergence than frozen eggs, with minimal or no sublethal effects on the progeny [37]. Therefore, in the present study, we adopted refrigerated eggs as a positive control. Refrigerated sterile eggs were obtained by keeping fresh eggs (<24 h since being laid) at 8 °C for 14 days, according to [37].

### 2.3. Egg Dose Response

An initial dose-response experiment for BMSB eggs was conducted in order to identify the dose of irradiation needed to induce complete egg sterility. In autumn 2021, fresh eggs from the rearing colony at the ENEA facilities in Rome were irradiated at doses of 16, 24, 32, and 40 Gy. The aging factor was also considered. Consequently, egg masses were irradiated 24 and 48 h after oviposition. Unirradiated eggs (0 Gy dose) of 24 and 48 h were used as the control. The number of replicates was approximately 10, with at least 6 replicates per treatment. After irradiation, eggs were placed in 9 cm diameter Petri dishes and were moved to a climatic chamber (27 ± 1 °C, photoperiod L.D. = 16:8, RH = 70 ± 10%) for 8 days, which is when all of the eggs should be virtually hatched, according to Nielsen et al. [42]. The number of hatched eggs per egg mass, the hatching rate (%) of eggs per egg mass, the number of immature embryos per egg mass, and their percentage were then all recorded with a Zeiss Stemi 508 KMAT stereomicroscope (10× magnification) (Zeiss, Oberkochen, Germany). Immature embryos were recorded regardless of subsequent egg hatching.

### 2.4. Male Dose Response

A second experiment was conducted to produce complete sterility of BMSB adult males in order to obtain sterile eggs by a conventional SIT approach. Male adult BMSBs from the ENEA rearing colony were irradiated ~24 h after emergence at doses of 24 and 50 Gy. Unirradiated males (0 Gy dose) were used as controls. After irradiation, males were moved to a climatic chamber (27 ± 1 °C, photoperiod L:D. = 16:8, RH = 70 ± 10%), and they were reared with newly emerged fertile females in cages (60 × 30 × 30 cm BugDorm^®^, Taichung, Taiwan) at a density of about 50–70 adults per cage with a 1:1 sex ratio. The eggs laid were collected daily and kept in 9-cm diameter Petri dishes for eight days until hatching. The same parameters were recorded as described above for irradiated eggs—the hatching rate (%) of eggs and the number of immature embryos in the eggs.

### 2.5. Evaluation of Sterile Eggs as an Oviposition Substrate

From March to June 2022, the BMSB, irradiated, and SIT eggs ≤ 24 h old were sent from ENEA to FEM using a courier, with delivery in 24 h. The egg masses were stored in Petri dishes secured in polystyrene boxes for transportation. The temperature inside the box was registered with a data logger (EL-USB-2, Lascar Electronics, Whiteparish, UK), and the values ranged between 18 °C and 23 °C. After delivery, egg masses (number of eggs per egg mass = 25–28) were glued with a drop of odourless polyvinyl acetate-based glue Vinavil^®^ (Vinavil, Milan, Italy) on a 2 × 8 cm piece of cardboard, and were then placed into a plastic tube (VWR 50-mL centrifuge tubes, 525–0611) with honey (with droplets placed on the inside of the screw cap). Each egg mass had been exposed to one 2-week-old *T. japonicus* mated female originating from the FEM rearing colony. The age of the females was chosen to maximize the ovary egg load without compromising their lifespan [43]. Moreover, these females were isolated in the plastic tube containing honey that provided an ad libitum food source for 24 h before being offered to the BMSB egg masses. Egg masses were maintained in a climatic chamber at a temperature of 24 ± 1 °C, photoperiod L:D = 16:8, and a relative humidity (RH) of 60 ± 5% until the emergence of *T. japonicus* (generally after 15 ± 2 days). To evaluate the suitability of BMSB eggs over time and their potential use in male sterile release programs and field monitoring of egg parasitoids, we kept the eggs at standard climatic conditions prior to their exposure to parasitism in tests (L:D = 16:8, T = 20 °C, RH = 60%). The eggs were placed in a climatic chamber with the abovementioned parameters for different periods: 4, 7, 10, 15, and 20 days before exposure to a *T. japonicus* female. Refrigerated eggs were used as a positive control following the protocol of [37]. Refrigerated eggs were placed in a climatic chamber, as with the other two types of sterile eggs, for different lengths of time: 1, 4, 7, 10, 15, and 20 days before their exposure to a *T. japonicus* ovipositing female. From here on, when we refer to the age of the eggs, we mean the elapsed time spent in the climatic chamber (L:D = 16:8, T = 20 °C, RH = 60%) before exposure to the parasitoid.

The number of replicates was approximately 30 per treatment (irradiated, SIT, and refrigerated) at different ages.

After 24 h, the female was removed from the vial with the egg mass, and the screw cap with honey was substituted with another without honey to avoid feeding the emerged individuals before recording the key parameters (i.e., the dry weight).

To evaluate the acceptance of eggs and the possible non-lethal impacts in the larval development of *T. japonicus* caused by sterile eggs, due to either direct irradiation or mating with sterile males, we measured the following parameters:

(a) The percentage of parasitoids that emerged on the total number of eggs.
(b) The sex ratio of the emerged parasitoids.
(c) The longevity of the emerged parasitoid females.
(d) The fecundity of the parasitoid females at 0 and 12 days after emergence.
(e) The female dry weight (in the F1 generation).

To estimate the longevity, one emerged *T. japonicus* female per egg mass was isolated in a plastic tube (VWR 50-mL centrifuge tubes, 525–0611) containing honey and folded blotting paper stapled to a small card as a shelter. The mortality was checked weekly, and the honey was refreshed when necessary (at least once a week).

To measure fecundity, we dissected *T. japonicus* female specimens at two intervals following their emergence (0 days and 12 days) to record the initial egg load (0 days, <24 h) and the maximum egg load (12 days post-emergence), according to [37]. To determine the egg count immediately after emergence (within 24 h), we employed a method involving rapid freezing (−20 °C) of each female specimen for 30 min. Subsequently, the female was preserved in a 1.7 mL microcentrifuge tube containing a solution of NaCl 0.9%. To record fecundity after 12 days, each female was isolated for 12 days in a plastic tube (VWR 50-mL centrifuge tubes, 525–0611) containing honey, and then processed as the 0-day fertility samples. Subsequently, by dissecting the abdomen of a female with two entomological pins under a stereomicroscope, it was possible to count the number of mature ovarioles.

Female dry body weight, serving as an indicator of body size, was assessed following the methodology outlined in [37]. Three females of *T. japonicus* were placed in a 1.7 mL microcentrifuge tube containing 75% ethanol. Subsequently, they were transferred to a glass chamber equipped with desiccant (silica gel) for a period of 48 h to facilitate drying. The desiccated parasitoids were then weighed with a Sartorius CP2P microbalance (Sartorius, Göttingen, Germany) to the nearest microgram (μg). The weight of three females from each egg mass was measured simultaneously, and the total weight was divided by three to determine the average dry weight for females from each egg mass.

Eggs still unhatched after two weeks were dissected to check for the presence of an undeveloped or unemerged larva.

### 2.6. Statistical Methods

Egg dose-response and male dose-response: Data analyses were performed using SPSS Inc. PASW Statistics 17.0 (Taylor and Francis Group, Hove and New York, USA) [44].

Egg Dose Response: Effects of gamma irradiation on the examined response variables (number of nymphs, the percentage of eggs hatching, and the number of immature embryos) were analysed by a Generalized Linear Model (GLM) [45,46]. Due to the strong overdispersion in the dataset due to both uneven variance across factor levels and non-normal frequency distributions, a negative binomial distribution was applied. Two factors were considered as explanatory variables in the analysis: the applied dose (0, 16, 24, 32, and 40 Gy) and the time elapsed from the oviposition up to the irradiation treatment (24 and 48 h). A model with a full factorial design was chosen. The dose Gy was treated as a covariate in the model. A minimum of six repetitions were performed. Dunn’s test with Bonferroni correction was used as a post hoc test.

Male Dose Response: For irradiated adults, the same response variables were analysed, and a simple GLM model with a negative binomial distribution and one factor as an explanatory variable (the dose Gy at 0, 24 and 50 Gy) was applied. In the case of the tests with irradiated adults, 10 repetitions for both 0 and 24 Gy doses were carried out, whereas 100 repetitions were made for the 50 Gy dose in order to more precisely estimate the frequency of egg eclosion and the survival of the juvenile forms when these values approached zero. Dunn’s test with Bonferroni correction was used as a post hoc test.

Evaluation of sterile eggs as an oviposition substrate: Data analysis and plotting were performed with R version 4.2.2 [47] and ggplot2 [48]. A Kruskal–Wallis test and a Dunn post hoc test with Bonferroni correction were used to compare the proportion of emergence, the sex ratio, the female dry weight, the longevity of the emerged females, and the fecundity (at 0 and 12 days) for the three treatments (I, R, S) according to the age of the exposed egg masses (1, 4, 7, 10, 15, or 20 days).

To determine if the interaction between treatment and egg mass age was significant, we used generalized additive models (GAMs), considering the non-linearity of the data [49]. For the proportion of emergence and the sex ratio, a binomial error distribution was applied, while the remaining parameters were analysed with a Poisson error distribution. The treatment (I, R, or S) was modelled as a factor (nominal variable), while a smoothing function was used for the egg mass age.

## 3. Results

### 3.1. Egg Dose Response

According to the GLM (Table 1), a strong negative effect of gamma irradiation was observed both on the development of the BMSB embryo and on the number of eggs hatching. In fact, only a few eggs hatched even at the lowest dose of irradiation of 16 Gy (Table 2). A statistically significant interaction was observed for egg age (24 or 48 h from collection) (Table 2), suggesting a differential effect of the irradiation on the eggs hatching, depending on the timing of the treatment. No egg hatch was observed when the irradiation was performed within the first 24 h after oviposition, while few cases of eggs hatching were observed at 48 h up to 32 Gy (Table 2).

### 3.2. Male Dose Response

The effect of irradiation on the adult males and their progeny was analysed at 24 and 50 Gy in comparison to the untreated control. As the data show, the effect of irradiation was strong on egg hatch rate according to the GLM (χ^2^ = 412.3, df = 1, *p* < 0.001), and was also strong on immature embryo formation (χ^2^ = 421.2, df = 1, p < 0.001). Very low values of egg hatch rates were seen at 50 Gy (0.81% ± 0.21 in comparison to 92.1% ± 4.39 of the untreated controls (0 Gy) (Table 3), while at the 24 Gy dose, the percentage of eggs hatching was intermediate (23.47 ± 6.1) between the untreated control and 50 Gy, and significantly different from the 0 and 50 Gy doses according to Dunn’s test.

### 3.3. Evaluation of Sterile Eggs as an Oviposition Substrate

**Figure 1:**
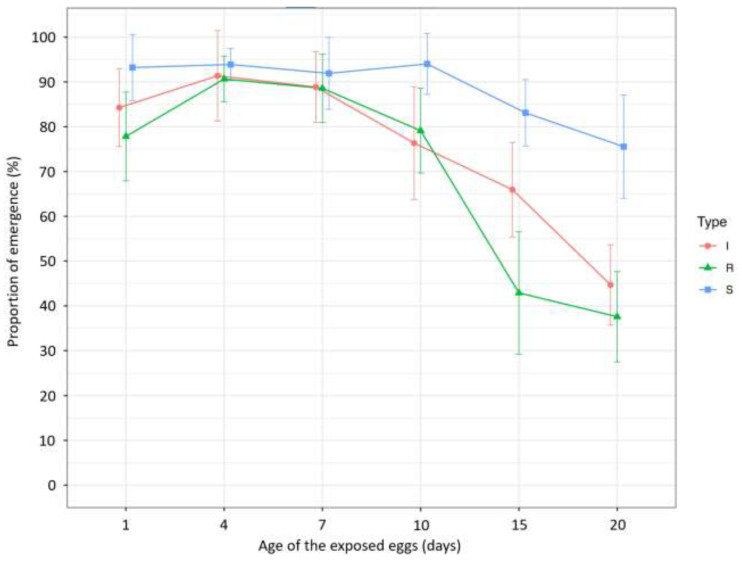
Average (±95% confidence interval) proportion of emergence (%) for the egg mass type (I, Irradiated; R, Refrigerated; S, SIT) at different egg age.

The proportion of emergence (%) of parasitoids had quite different trends for the three egg types (Figure 1, Table 4). The 1-day-old SIT eggs emerged at rates above 90% and continued with similar values at 4, 7, and 10 days of age. At 15 and 20 days, the proportion of emerged insects dropped to 83.12% and 75.56%, respectively. In contrast, irradiated and refrigerated eggs had lower emergence values for 1-day-old eggs (84.28% and 77.85%), while values were higher at 4 days (91.39% and 90.66%) and remained at similar values even at 7 days (88.86% and 88.58%), and, statistically, they did not differ significantly from the SIT eggs of 4 and 7 days. From 10 to 20 days of the age of the egg, the proportion of emergence of both the irradiated and the refrigerated eggs dropped significantly compared to the SIT eggs.

The sex ratio, expressed as the percentage of the emerged females of the total number of the emerged parasitoids (Table 4), was significantly higher in the 1-day-old SIT eggs (88.34%) compared to the irradiated (84.28%) and the refrigerated ones (78.48%). For eggs aged 4 and 7 days, the parasitoid emergence rate was similar for all three types of egg (the values varied between 81.45% and 89.25%), while it dropped significantly in 10-day-old eggs for the irradiated (61.79%) and the refrigerated types (73.82%) when compared to the SIT ones (91.95%). With the increase in the age of the BMSB eggs, the decline in the percentage of emerging parasitoid females was greater for the irradiated and the refrigerated eggs than it was for the SIT ones. Indeed, for the 20-day-old eggs, the emergence rate of *T. japonicus* females was 40.55% for the irradiated ones, 39.67% for the refrigerated ones, and 73.45% for the SIT type (Table 4). Possible sub-lethal effects on the progeny of *T. japonicus* due to larval development in sterile eggs were evaluated by measuring the fecundity, the dry weight, and the longevity in the emerged parasitoids.

The fecundity at initial egg load (Fo) did not differ among the three categories of eggs (1, 4, and 10 days old), whereas at 7, 15, and 20 days, fecundity was significantly lower for the irradiated eggs than it was for the refrigerated and the SIT ones (Table 4). On the other hand, fecundity at maximum egg load (F12) was constant for the SIT eggs, which also showed the highest values for almost all of the egg ages. The values of the other two egg types were rather variable (Table 4).

The dry weight was constant for the SIT eggs, which showed the highest values for each age group of eggs. The other two treatments of eggs both showed highly variable values among the different ages—sometimes similar to the SIT eggs and sometimes significantly different (Table 4).

Regarding longevity (expressed in days), the only statistically significant differences were observed in the progeny from 7-day-old and 20-day-old eggs. In both cases, the highest values were observed for irradiated eggs (Table 4).

**Figure 2:**
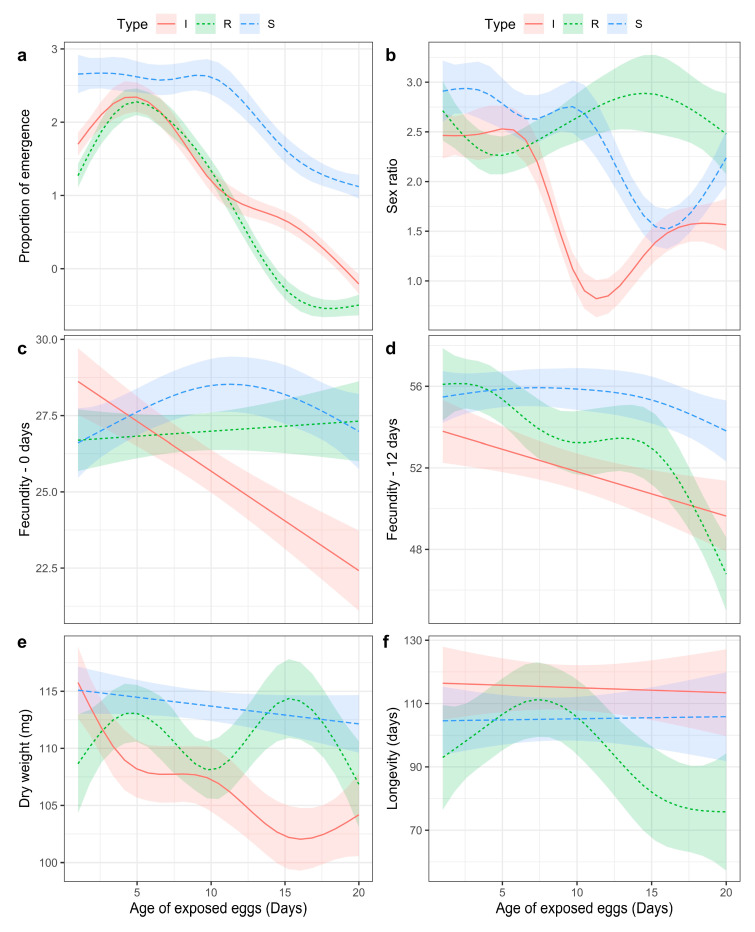
Estimated smooth functions for the generalized additive models (GAMs), according to egg mass type (I, Irradiated; R, Refrigerated; S, SIT). Smoothing parameters estimated using restricted maximum likelihood (REML). Different letters indicate different parameters: (**a**), Proportion of emergence; (**b**), Sex ratio; (**c**), Fecundity—0 days; (**d**), Fecundity—12 days; (**e**), Dry weight; (**f**), Longevity.

The estimated smoothing curves for the GAMs and the factor variables (Figure 2) supported a non-linear relationship between the recorded parameters and the age of the egg mass, which varied with the treatment. A backwards selection using the Akaike Information Criteria indicated that no terms should be dropped from the models. According to egg mass type, the analysis confirmed the significant influence of the age of the eggs for the parameters considered (Table 5).

Regarding the proportion of the emergence (%) of parasitoids, the SIT eggs showed an initial plateau, and no significant decrease was observed up to 10 days. On the other hand, for the irradiated and the refrigerated eggs, the proportion of emergence was inversely proportional to the percentage of hatching immediately, and it increased on the fifth day when the highest proportion of emergence was recorded. After that, it decreased significantly (Figure 2a; Table 5).

The sex ratio (%) was also influenced by the age of the eggs (Table 5). The decrease in the proportion of females was more influenced by the egg age for the irradiated and SIT eggs than for the refrigerated ones (Figure 2b). Specifically, a significant decline in sex ratio was observed after 5 days for the irradiated eggs and after 10 days for the SIT ones, while for the refrigerated eggs, the influence of the age of the egg was less significant compared to the other two types (Table 5). The pattern observed in the refrigerated eggs exhibited considerable variability, with a decline at 5 days followed by a subsequent increase.

The fecundity at F0 was strongly influenced by the age of the eggs for the irradiated eggs. The same influence was slightly significant for the SIT eggs, while it was not significant for the refrigerated eggs (Figure 2c; Table 5).

The fecundity at F12 was significantly influenced by the age of the egg for the irradiated and the refrigerated eggs, and declined as the eggs aged. The SIT eggs were not influenced by egg age for this parameter (Figure 2d; Table 5); indeed, the load of the ovarial eggs was constant, independently of the age of the eggs (Table 4).

The dry weight declined significantly for the irradiated and refrigerated eggs, as influenced by egg age. The strongest decline was observed for the irradiated eggs at 5 days, while the SIT eggs were not influenced by egg age and showed a linear trend (Figure 2e; Table 5). Longevity was not affected by the age of the egg for the irradiated and the SIT eggs, which both showed a linear trend, while it decreased significantly for the refrigerated eggs (Figure 2f; Table 5).

## 4. Discussion

An effective pest management program based on CBC requires the release of an appropriate number of parasitoids (or other natural enemies) suitable for the establishment of a self-sustaining population of the biocontrol agent while reducing the population of the target pest [50].

Sterile eggs laid by wild females after mating with irradiated males can play an important role in the management of a BMSB population, either to facilitate and accelerate the natural multiplication of the released oophagous biocontrol agent or as sentinel eggs for field monitoring.

For this purpose, sterile eggs obtained by the irradiation of fresh eggs could also play a role in the multiplication and the field monitoring of the egg parasitoids of the target species.

However, until now, BMSBs as well as other pentatomid bug species have not been considered as targets for the classic SIT approach because mass rearing studies so far have mainly addressed holometabolous insect orders, such as Diptera and Lepidoptera. Moreover, sterile BMSB adults can damage plants by their feeding, as their piercing-sucking mouthparts can cause unacceptable damage to cultivated fruits and vegetables. Given the favourable results with all of the evaluated parameters, establishing a small-scale SIT laboratory colony of BMSBs in order to produce large numbers of SIT eggs could be considered a suitable approach for pre-release parasitoid multiplication or for their field monitoring. Cristofaro et al. [38] suggested the use of SIT-sterilized eggs for a new concept of “sterile sentinel eggs”, in which SIT eggs should be more attractive than frozen eggs because they are less physiologically manipulated. This concept could explain the difference that was observed between the SIT eggs and the other two types in this study, for which manipulations after oviposition (irradiation or refrigeration) were required to reach sterility. Indeed, since egg parasitoid females use kairomone cues for short-range host locations [51,52], direct irradiation and refrigeration of the eggs could affect chemical traces (footprints) left by the BMSB females. Semiochemical interactions between sterile eggs (SIT, irradiated, and refrigerated) and egg parasitoids need further investigation.

Nonetheless, if managed well, refrigerated and unfertilized eggs produced by unmated females can be successfully used for field monitoring and for rearing colonies [36,37]. Indeed, refrigerated eggs are easier to obtain and store for relatively long periods (two months in a refrigerator at 8 °C) before the emergence of *T. japonicus* decreases significantly [37]. In our experiment, the data showed that during the first week, the performance of the BMSB refrigerated eggs as a suitable substrate for *T. japonicus* oviposition and larval development was similar to the SIT eggs, which confirms the data reported by [37] (Figure 1, Table 4). On the other hand, unfertilized eggs were also suitable for both parasitization and larval development of *T. japonicus* for up to 11 days [36]. In this respect, irradiated eggs, which were less suitable than SIT eggs and no better than refrigerated eggs (for most of the parameters evaluated), are not recommended for monitoring, considering that a BMSB female may produce multiple egg masses during her lifetime [2,36,42] and that the production of SIT eggs or one of the other abovementioned alternatives are less time-consuming and also cheaper.

The results from the screening to assess egg sterility of BMSBs by irradiation of fresh eggs (maximum 48 h) showed that no eggs hatched when the irradiation was performed at 24 h, while sporadic cases of egg eclosion were observed at 48 h up to 32 Gy (Table 2). However, in many cases, we observed immature and malformed embryos, which are not a good substrate for the parasitoid’s larval development [53] (Table 2), particularly at 16 Gy (30.95% at 24 h and 25% at 48 h) and at 24 Gy (30.36% at 24 h, and then 0% at 48 h). The 40 Gy dose did not show any egg hatching or any development of embryos, and it was thus chosen as the optimal dose for exposure to *T. japonicus* and is probably suitable for the egg sentinel technique.

For the male irradiation response, at 50 Gy, immature embryo formation was sporadic (3.86%) compared to 24 Gy (31.55%) and the percentage of eggs hatched was 0.81% compared with 23.47% at 24 Gy (Table 3), which indicates that 50 Gy was a reliable oviposition substrate for egg parasitoids and potentially suitable for their use as egg sentinels. Even if a few SIT eggs hatched (at 50 Gy), as reported in other studies on BMSB irradiation, the progeny of irradiated males exhibit high mortality [54,55,56], and cumulative sterility has been observed when individuals born from incompletely sterile egg masses reach the adult stage [55,56]. Consequently, the poor hatching rate observed at 50 Gy does not represent a problem for implementing SIT, despite the partial sterility reported [57], which was over 99% in our study.

The exposure of sterile BMSB egg masses at different ages to *T. japonicus* showed that the SIT eggs remained a suitable substrate for oviposition up to 20 days under standard experimental conditions (L:D = 16:8, T = 20 °C, RH = 60%) and also under the subsequent conditions adopted for larval development (L:D =16:8; T = 24 °C; RH = 60%) (Figure 1, Table 4). On the other hand, the irradiated and refrigerated eggs showed a higher proportion of emergence in 4-day-old eggs compared to 1-day-old ones, contrary to the expectation (Figure 1, Table 4). We presume that the exposure of the eggs to the preoviposition conditions is responsible for these data, but further investigations will be needed to establish the mechanism behind this.

Interestingly, none of the SIT eggs hatched or developed embryos during exposure at a 20 °C constant temperature before being exposed to *T. japonicus,* while some development partially occurred at 27 °C (Table 3). These data are also unexpected, considering that [42] reported an average development time of 11.5 days at a constant 20 °C temperature exposure with only a 1% rate of egg mortality. The data suggest the occurrence of a possible inhibition of the development of *H. halys* embryos in SIT eggs kept at 20 °C, with complete sterility as a result.

In the SIT eggs, the sex ratio of the progeny was slightly influenced by the age of the eggs compared to the irradiated and refrigerated eggs (Table 4), and this suggests that the SIT eggs remain a better substrate for the larval development of *T. japonicus* than the other two types.

However, the influence of the age of the egg on the reduction of the emerging females (%) was statistically significant (Figure 2; Table 5). Fecundity (both F0 and F12) and dry weight both confirmed that the SIT eggs were, in general, less influenced by egg age (Table 5), and that they showed the highest values of egg load and dry weight among the treatments, especially when the age of the egg was more than 10 days (Table 4). The age of the egg did not influence the longevity of the progeny that emerged from the SIT and irradiated eggs, while the progeny from the refrigerated eggs was significantly influenced (Table 5). Notably, parasitoids that developed in the 20-day-old refrigerated eggs showed a significant reduction in longevity (Table 4).

The differences observed among the treatments could also depend on the differences between the nutrient content of the eggs. Skillman and Lee 2017 [58] reported alterations in nutrient content related to the length of time that the eggs were frozen. In a similar way, the nutrient content of irradiated and refrigerated eggs might be differently altered by the process of sterilization for SIT eggs, where egg sterility is induced by the irradiation of the male since the eggs are not exposed to direct γ irradiation or refrigeration. Nonetheless, our data confirm that refrigerated eggs are a good substrate for the oviposition and the larval development of *T. japonicus* up to 7 days without any negative effects on the parameters evaluated. The decreasing female offspring (sex ratio) observed in our experiments correspond with the data reported on unfertilized eggs [36], showing a significant decrease in terms of sex ratio when exposed to seven day or older host eggs. This highlighted a loss of quality as an oviposition substrate for all of the types of eggs that we tested as the egg age increases, and this is similar to the results reported for fresh eggs [53].

In a previous study [54], we assessed the suitability of overwintering males for the Sterile Insect Technique (SIT) as a potential source of irradiated wild males in an IPM strategy. To implement an area-wide IPM (AW-IPM) or eradication program for managing the BMSB species in its invasive range, we considered the complementary effects that can be achieved through a combination of SIT and classical biological control methods [25,26]. Thus, in addition to reducing the population density of the target species as a suppression method [24], the outcome of an SIT approach—sterile eggs laid by wild females mated with irradiated males—could provide a valid oviposition resource, which would be useful for enhancing both the establishment and the multiplication of the selected egg parasitoids. In addition, sterile eggs derived by SIT could have an alternative role in field monitoring or in supporting rearing colonies.

A complementary effect deriving from the combined use of SIT and biological control [29,59] was considered for the suppression or eradication of a target species. In previous studies [60,61], the combined release of both sterile BMSB males and egg parasitoids was proposed as an alternative management strategy. However, this approach, even though it may be a more environmentally friendly solution (assuming there are no offspring from either the released target or from its parasitoid), does not provide the possibility for sterile eggs to multiply parasitoids. Indeed, as mentioned before, SIT eggs have the potential to be a suitable oviposition substrate for *T. japonicus* multiplication, readily available in the field after mating between sterile BMSB males with wild females. In this sense, the present study provides the foundation for a potential control tactic that involves the use of SIT and biocontrol for BMSBs by releasing sterile BMSB males. Egg predation would remain a concern, though, with extended field exposure of SIT eggs in terms of egg predation. We expect that SIT eggs will be subjected to predation just as much as fresh and frozen sentinel eggs [32,62], but they will nonetheless be available to egg parasitoids for longer periods of time than fresh or frozen eggs [37]. The results obtained in this study therefore provide a valuable starting point for implementing a new strategy that combines SIT and biocontrol for the BMSB species. Further field and semi-field tests are still needed, though, to assess the real feasibility of this potential IPM strategy.

## Figures and Tables

**Table 1:** Effects of gamma irradiation on *Halyomorpha halys* egg masses at 24 and 48 h after egg collection.

	n. of Visible Embryos		n. of Hatched Eggs	
	χ^2^	*p*-Value	χ^2^	*p*-Value
Intercept	46.186	1.1 × 10^−11^	67.274	2.4 × 10^−16^
Gy	28.514	9.3 × 10^−8^	44.762	2.2 × 10^−11^
hours	0.641	0.423	0.091	0.763
Gy × ours	0.480	0.488	7.662	5.6 × 10^−3^

**Table 2:** Effects of gamma irradiation on *Halyomorpha halys* egg masses irradiated at 24 h and 48 h after egg collection. The data refer to a period of 6–8 days after oviposition, when virtually all eggs were hatched, and only the first instar nymphs that were still alive were considered in the eggs hatched.

Dose (Gy)	Egg Age (hours)	*n*	Immature Embryos		Eggs Hatched	
			(n.) ^1^	(%) ^1^	(n.) ^1^	(%) ^1^
0	24	10	25.67 ± 1.48 a	91.67 ± 5.29 a	21.80 ± 2.74 a	77.86 ± 9.80 a
0	48	6	26.00 ± 1.53 a	92.86 ± 5.46 a	24.67 ± 1.86 a	88.10 ± 6.63 a
16	24	12	9.77 ± 2.58 b	30.95 ± 5.83 b	0.00 b	0.00 b
16	48	10	7.00 ± 3.69 bc	25.00 ± 13.17 bc	0.60 ± 0.43 b	2.14 ± 1.52 b
24	24	14	8.50 ± 3.11 b	30.36 ± 11.10 b	0.00 b	0.00 b
24	48	10	0.00 c	0.00 c	1.50 ± 1.50 b	5.36 ± 5.36 b
32	24	12	0.00 c	0.00 c	0.00 b	0.00 b
32	48	24	2.79 ± 1.56 c	9.97 ± 5.56 c	2.04 ± 1.39 b	9.07 ± 5.20 b
40	24	10	0.00 c	0.00 c	0.00 b	0.00 b
40	48	10	0.00 c	0.00 c	0.00 b	0.00 b

^1^ Mean ± se; different letters indicate significant differences (*p* < 0.05) in the same column, according to a Dunn’s test with Bonferroni correction.

**Table 3:** Immature embryos (visible embryos after oviposition) and eggs hatched after 6 days from the oviposition of the SIT eggs.

Dose (Gy)	*n*	Immature Embryos		Eggs Hatched	
		n. ^1^	% ^1^	n. ^1^	% ^1^
0	10	26.50 ± 0.582 a	95.96 ± 1.63 a	25.50 ± 1.327 a	92.14 ± 4.39 a
24	10	8.83 ± 1.662 b	31.55 ± 5.93 b	6.57 ± 1.702 b	23.47 ± 6.08 b
50	100	1.08 ± 0.176 c	3.86 ± 0.63 c	0.23 ± 0.060 c	0.81 ± 0.21 c

^1^ Mean ± se; different letters indicate significant differences (*p* < 0.05) in the same column according to Dunn’s test with Bonferroni correction.

**Table 4:** Mean and standard deviation for the different parameters, according to the age of the exposed egg mass. Significant differences verified with the Kruskal–Wallis test (H) and a post hoc Dunn’s test with Bonferroni correction, where letters indicate significant differences (*p* < 0.05).

Parameter	Age (days)	H	df	*p*-Value	Irradiated	Refrigerated	SIT
					Mean ± sd ^1^	Mean ± sd ^1^	Mean ± sd ^1^
Proportion of emergence	1	17.591	2	<0.001	84.28 ± 27.9 b	77.85 ± 27 c	93.23 ± 20.12 a
	4	4.464	2	0.107	91.39 ± 25.49	90.66 ± 14.58	93.90 ± 9.45
	7	5.777	2	0.056	88.86 ± 21.23	88.58 ± 20.84	91.93 ± 23.4
	10	15.244	2	<0.001	76.36 ± 34.38 b	79.13 ± 27.51 b	94.03 ± 18.23 a
	15	19.226	2	<0.001	65.96 ± 32.1 b	42.9 ± 35.9 c	83.12 ± 19.82 a
	20	25.284	2	<0.001	44.68 ± 24.9 b	37.57 ± 27.49 b	75.56 ± 30.25 a
Sex ratio	1	13.791	2	0.001	81.66 ± 24.06 b	78.48 ± 18.15 c	88.34 ± 19.95 a
	4	2.460	2	0.292	84.20 ± 25.95	81.45 ± 25.15	89.25 ± 10.45
	7	7.664	2	0.022	84.11 ± 20.44	84.15 ± 17.19	87.61 ± 22.79
	10	19.095	2	<0.001	61.79 ± 38.59 c	73.82 ± 26.47 b	91.95 ± 5.28 a
	15	4.210	2	0.122	60.37 ± 31.42 b	53.85 ± 28.8 b	68.56 ± 32.45 a
	20	24.839	2	<0.001	40.55 ± 21.95 b	39.67 ± 23.26 b	73.45 ± 26.61 a
Fecundity—0 days	1	3.748	2	0.154	28.82 ± 4.19	26.14 ± 1.66	26.58 ± 3.36
	4	1.851	2	0.396	27.7 ± 2.99	26.31 ± 2.92	27.12 ± 3.60
	7	7.726	2	0.021	23.5 ± 5.43 b	28.95 ± 3.33 a	28.15 ± 3.52 a
	10	3.520	2	0.172	28.5 ± 3.03	26.48 ± 3.65	28.78 ± 2.97
	15	12.207	2	0.002	23.59 ± 4.58 b	26.36 ± 1.96 ab	28.35 ± 2.31 a
	20	24.363	2	<0.001	22.43 ± 1.79 b	27.47 ± 2.07 a	26.75 ± 2.63 a
Fecundity—12 days	1	2.283	2	0.319	53.78 ± 3.38	55.78 ± 3.28	55.21 ± 4.24
	4	2.556	2	0.279	53.89 ± 4.19 b	56.33 ± 3.2 a	55.96 ± 4.28 ab
	7	16.595	2	<0.001	49.5 ± 3.67 b	53.63 ± 3.52 b	56.29 ± 3.11 a
	10	2.492	2	0.288	53.06 ± 3.25	52.78 ± 3.08	55 ± 4.22
	15	17.751	2	<0.001	49 ± 3.14 b	53.75 ± 3.72 a	56.63 ± 3.89 a
	20	5.646	2	0.059	50.83 ± 1.8 ab	46.53 ± 8.64 b	53.25 ± 3.05 a
Dry weight	1	7.499	2	0.024	116.57 ± 11.98 a	108 ± 5.87 b	115.98 ± 8.04 a
	4	10.717	2	0.005	107.59 ± 5.65 b	113.41 ± 6.22 a	112.65 ± 8.96 a
	7	14.239	2	0.001	107.87 ± 7.57 b	111.65 ± 4.51 ab	115.68 ± 5.89 a
	10	7.715	2	0.021	108.54 ± 7.56 ab	107.26 ± 9.6 b	113.6 ± 8.12 a
	15	21.866	2	<0.001	101.57 ± 7.77 b	115.25 ± 13.13 a	112.12 ± 7.85 a
	20	14.879	2	0.001	104.57 ± 8.65 b	106.51 ± 4.83 b	112.64 ± 5.23 a
Longevity	1	2.898	2	0.235	115.75 ± 44.54	93.41 ± 55.16	106.2 ± 44.26
	4	1.504	2	0.471	109 ± 48.67	97.84 ± 39.43	96.21 ± 43.19
	7	6.399	2	0.041	133.64 ± 36.1 a	121.67 ± 39.51 ab	105.5 ± 49.97 b
	10	1.539	2	0.463	107.67 ± 53.8	107.35 ± 34.85	116.24 ± 40.19
	15	4.877	2	0.087	104.9 ± 39.58	74.58 ± 50.52	101.65 ± 39.3
	20	11.127	2	0.004	120.46 ± 41.53 a	78.9 ± 45.54 b	103.22 ± 43.8 ab

^1^ Different letters indicate significant differences in the same row.

**Table 5:** Summary table of GAMs results. Egg mass type: irradiated (I), refrigerated (R), and SIT (S).

Model	Adjusted R^2^	Deviance Explained	Component	Term	Estimate	Std Error	t-Value	*p*-Value
Proportion of emergence	30.70%	29%	A. Parametric coefficients	(Intercept)	1.290	0.037	34.993	<0.001
				Type R	−0.272	0.052	−5.265	<0.001
				Type S	0.927	0.062	14.863	<0.001
			B. Smooth terms	s(Day):Type I	4.536	4.867	625.785	<0.001
				s(Day):Type R	4.655	4.926	926.123	<0.001
				s(Day):Type S	4.005	4.521	212.335	<0.001
Sex ratio	6.93%	14%	A. Parametric coefficients	(Intercept)	1.886	0.049	38.636	<0.001
				Type R	0.678	0.085	8.010	<0.001
				Type S	0.630	0.077	8.224	<0.001
			B. Smooth terms	s(Day):Type I	4.731	4.955	169.552	<0.001
				s(Day):Type R	3.644	4.293	11.964	0.025
				s(Day):Type S	4.479	4.852	96.429	<0.001
Fecundity—0 days	13.70%	15%	A. Parametric coefficients	(Intercept)	25.966	0.355	73.043	<0.001
				Type R	0.992	0.470	2.109	0.036
				Type S	1.652	0.448	3.684	<0.001
			B. Smooth terms	s(Day):Type I	1.001	1.001	38.540	<0.001
				s(Day):Type R	1.000	1.000	0.383	0.536
				s(Day):Type S	2.347	2.872	2.438	0.057
Fecundity—12 days	26.40%	28%	A. Parametric coefficients	(Intercept)	52.019	0.457	113.917	<0.001
				Type R	1.317	0.592	2.224	0.027
				Type S	3.373	0.565	5.972	<0.001
			B. Smooth terms	s(Day):Type I	1.003	1.006	8.767	0.003
				s(Day):Type R	3.624	4.224	17.204	<0.001
				s(Day):Type S	2.073	2.540	1.984	0.102
Dry weight	19.50%	22%	A. Parametric coefficients	(Intercept)	107.723	0.687	156.807	<0.001
				Type R	2.764	0.993	2.783	0.006
				Type S	6.092	0.951	6.405	<0.001
			B. Smooth terms	s(Day):Type I	3.689	4.275	10.147	<0.001
				s(Day):Type R	4.209	4.705	3.543	0.004
				s(Day):Type S	1.000	1.000	2.335	0.127
Longevity	4.59%	6%	A. Parametric coefficients	(Intercept)	115.147	3.612	31.875	<0.001
				Type R	−19.087	5.082	−3.755	<0.001
				Type S	−10.033	4.999	−2.007	0.045
			B. Smooth terms	s(Day):Type I	1.000	1.001	0.079	0.779
				s(Day):Type R	2.995	3.613	3.946	0.009
				s(Day):Type S	1.003	1.006	0.015	0.913

## Data Availability

The data presented in this study are available on request from the authors.
